# A letter to editor in response to the article on management of patients with sickle cell disease undergoing valve surgery in Sudan

**DOI:** 10.1093/icvts/ivad033

**Published:** 2023-02-14

**Authors:** Sulafa K M Ali

**Affiliations:** Department of Pediatric Cardiology, Sudan Heart Center, University of Khartoum, Khartoum, Sudan

**Keywords:** Sickle cell disease, Valve surgery, Sudan

Dear Editors

I read with interest the article by Epis *et al.* [1], reporting on management of patients with sickle cell disease (SCD) undergoing valve surgery.

Regarding diagnosis of SCD, the authors mentioned that ‘confirmed SCD patients who are anaemic with a suggestive medical history, plus a positive SCD test’ to indicate the methods of diagnosis of SCD. This statement indicated that they did not perform a haemoglobin electrophoresis test, which is the gold standard for diagnosis of SCD. Therefore, they probably included patients with sickle cell trait who typically have a milder form of disease. In the ‘limitations’ section, authors mentioned that there is limited availability of SCD laboratory investigations while in fact the tests are widely available and used routinely in Sudan [2, 3]. Furthermore, authors did not mention that they consulted haematologists to plan the perioperative management. This policy could have been acceptable if there is no haematology consultants available in the country; however, paediatric as well as adult haematologists are available in Khartoum and it is routine to consult them for preoperative assessment and management of patients with SCD. Although there is no consensus on preoperative blood transfusion policy (simple versus exchange transfusion), stratifying patients’ risk for SCD-related complications could have an impact on deciding blood transfusion policy. An important issue is ethical considerations, the authors obtained the Medical Ethics Clearance for this research from ‘Research Ethical Committee of the University of Milan—Italy’ despite that the patients were recruited from Sudan where Salam Center is located. This fact stands in sharp contrast to the Ethics of Medical Research. We believe that Research Ethics Committees are currently the standard of care for medical institutions.

In conclusion, the article has important methodological and ethical limitations. Collaboration will definitely improve patients’ care, which is the main objective of Salam Center and Emergency organization.

## References

[1] Epis F , Chatenoud L, Somaschini A, Bitetti I, Cantarero F, Salvati AC et al Simple open-heart surgery protocol for sickle-cell disease patients: a retrospective cohort study comparing patients undergoing mitral valve surgery. Interdisc CardioVasc Thorac Surg 2022;35:ivac205. 10.1093/icvts/ivac205 PMC9426665 36018254

[2] Daak AA , Elsamani E, Ali EH, Mohamed FA, Abdel-Rahman ME, Elderdery AY et al Sickle cell disease in western Sudan: genetic epidemiology and predictors of knowledge attitude and practices. Trop Med Int Health 2016;21:642–53. 27028397 10.1111/tmi.12689 PMC10699227

[3] Talha M , Osman B, Abdalla S, Mirghani H, Abdoon I. Pediatric sickle cell disease in Sudan: complications and management. Anemia 2022;2022:3058012. 35198244 10.1155/2022/3058012 PMC8860554

